# EBV encoded miRNA BART8-3p promotes radioresistance in nasopharyngeal carcinoma by regulating ATM/ATR signaling pathway

**DOI:** 10.1042/BSR20190415

**Published:** 2019-09-13

**Authors:** Xiaohan Zhou, Jialing Zheng, Ying Tang, Yanling lin, Lingzhi Wang, Ye Li, Chengdong Liu, Dehua Wu, Longmei Cai

**Affiliations:** 1 Department of Radiation Oncology, Nanfang Hospital, Southern Medical University, Guangzhou, China; 2 Second Clinical Medical College, Zhujiang Hospital, Southern Medical University, Guangzhou, China; 3 Department of Obstetrics and Gynaecology, Zhujiang Hospital, Southern Medical University, Guangzhou, China; 4 Department of Plastic and Cosmetic Surgery, Nanfang Hospital, Southern Medical University, Guangzhou, China

**Keywords:** ATM/ATR, DSBs, EBV-miR-BART8-3p, NPC, radioresistance

## Abstract

Resistance to radiotherapy is one of the main causes of treatment failure in patients with nasopharyngeal carcinoma (NPC). Epstein-Barr virus (EBV) infection is an important factor in the pathogenesis of NPC, and EBV-encoded microRNAs (miRNAs) promote NPC progression. However, the role of EBV-encoded miRNAs in the radiosensitivity of NPC remains unclear. Here, we investigated the effects of EBV-miR-BART8-3p on radiotherapy resistance in NPC cells *in vitro* and *in vivo*, and explored the underlying molecular mechanisms. Inhibitors of ataxia telangiectasia mutated (ATM)/ataxia telangiectasia mutated and Rad3-related (ATR) (KU60019 and AZD6738, respectively) were used to examine radiotherapy resistance. We proved that EBV-miR-BART8-3p promoted NPC cell proliferation in response to irradiation *in vitro* and associated with the induction of cell cycle arrest at the G2/M phase, which was a positive factor for the DNA repair after radiation treatment. Besides, EBV-miR-BART8-3p could increase the size of xenograft tumors significantly in nude mice. Treatment with KU60019 or AZD6738 increased the radiosensitivity of NPC by suppressing the expression of p-ATM and p-ATR. The present results indicate that EBV-miR-BART8-3p promotes radioresistance in NPC by modulating the activity of ATM/ATR signaling pathway.

## Introduction

Nasopharyngeal carcinoma (NPC) is a common malignant tumor in Southern China, although a high incidence is also reported in Southeast Asia, North Africa, Alaska and the Mediterranean basin [1]. NPC is highly radiosensitive, and radiotherapy with or without chemotherapy is the mainstay treatment [2]. The local control rate and 5-year overall survival of patients with NPC exceed 90 and 80%, respectively^3^]. Although technological advances have improved the prognosis of NPC, radioresistance remains the main cause of therapy failure and distant metastasis [4]. A better understanding of the mechanism underlying radioresistance of NPC may improve survival and facilitate design of therapeutic strategies.

Several factors are involved in the etiology of NPC; among these, Epstein-Barr virus (EBV) infection plays a central role [5]. EBV is the first human virus shown to encode miRNAs; indeed, 25 EBV-miRNA precursors containing 48 mature miRNAs have been identified within two regions of the EBV genome [6,7]. The BamHI fragment H rightward reading frame 1 (BHRF1) gene encodes three miRNA precursors (EBV-miR-BHRF1–3) that generate four mature miRNAs, whereas the BamHI fragment A rightward transcript (BART) region contains 22 miRNA precursors (EBV-miR-BART1–22) that produce 44 mature miRNAs [8]. EBV-encoded miRNAs promote migration and proliferation and inhibit apoptosis of NPC cells [9,10]. Proliferation and apoptosis of NPC cells in response to irradiation (IR) are an important factor determining radiosensitivity of NPC, which is important for clinical purposes. However, it is unclear whether EBV-encoded miRNAs are involved in progression and radiosensitivity of NPC.

MiRNAs modulate cell radiosensitivity by targeting specific DNA repair factors [11]. DNA repair is regulated by ataxia telangiectasia mutated (ATM) and ataxia telangiectasia mutated and Rad3-related (ATR) signaling pathway[12]. ATM and ATR are down-regulated in EBV-positive nasopharyngeal epithelial cells and primary NPC samples, ATM/ATR kinase activity followed by exposure to ionizing radiation [13,14]. The relationship between EBV-encoded miRNAs and ATM/ATR signaling pathway was investigated recently.

To the best of our knowledge, the present study is the first to show that EBV-miR-BART8-3p contributes to radioresistance in NPC by modulating ATM/ATR activity in response to DNA double-strand breaks (DSBs). These findings provide new insight into EBV-regulated radioresistance of NPC and may facilitate design of new treatment strategies.

## Materials and methods

### Cell culture and inhibitors

EBV-negative NPC cell lines (HONE1 and 5-8F) were obtained from the Cancer Research Institute, Southern Medical University. The EBV-positive NPC cell line HONE1-EBV was kindly provided by Professor S.-W. Tsao, University of Hong Kong. NPC cells were cultured in RPMI-1640 (Invitrogen) supplemented with 10% newborn cow serum (Hyclone, Invitrogen) at 37°C, 5% CO_2_. The highly specific and potent ATM/ATR inhibitors KU60019 and AZD6738 were purchased from Selleck Chemicals (Houston, TX, U.S.A.) and dissolved in 100% dimethyl sulfoxide (DMSO) before storage at −80°C.

### qRT-PCR

Total RNA was extracted using the TRIzol reagent (Invitrogen), and complementary DNA (cDNA) was synthesized with the PrimeScript RT reagent Kit (TaKaRa, Dalian, China). qRT-PCR was performed in triplicate with SYBR Premix ExTaq (TaKaRa). The primer used for amplification of Ebv-miR-BART8-3p was 5′-GTCACAATCTATGGGGTCGTAGA-3′. RPU6B (3′-CTCGCTTCGGCAGCACATATA-3′) was used for normalizing expression of miRNA. The fold changes were calculated using the 2^−ΔΔ*C*^_t_ method.

### Preparation of miRNA mimics or inhibitors and cell transfection

Cells were transfected with miRNA mimics or inhibitors (miRNA antisense oligonucleotides) at 50 nmol/l using Lipofectamine 2000 (Invitrogen). The EBV-miR-BART8-3p mimic (5′-GUCACAAUCUAUGGGGUCGUAGA-3′), EBV-miR-BART8-3p inhibitor (5′-UCUACGACCCCAUAGAUUGUGAC-3′), and associated nonspecific mimic (5′-UUGUACUACACAAAAGUACUG-3′) or inhibitor (5′-CAGUACUUUUGUGUAGUACAA-3′) controls were synthesized by GenePharma (Shanghai, China). 48 h post-transfection, the cells were harvested for qRT-PCR (Supplementary Figure S1A,B).

### Lentivirus transduction

Lentiviral particles containing the GV369 expression vector encoding the pri-EBV-miR-BART8 precursor, which produces BART8-5p and BART8-3p (Ubi-MCS-SV40-EGFP-IRES-puromycin-BART8), and a randomized flanking sequence control (Ubi-MCS-SV40-EGFP-IRES-puromycin-mork), were purchased from GeneChem (Shanghai, China) and transduced into NPC cells according to the manufacturer’s instructions. Virus-infected cells were GFP-positive (Supplementary Figure S2A,B).

### Acridine orange (AO) and ethidium bromide (EB) double staining

The DNA binding dyes acridine orange (AO) and ethidium bromide (EB) (Sigma Aldrich, U.S.A.) were used for morphological detection of apoptotic and necrotic cells. The cells were detached, washed with cold PBS, and stained with a mixture of AO (100 μg/ml) and EB (100 μg/ml) at room temperature for 5 min. Stained cells were visualized using a fluorescence microscope (Leica DM 3000, Germany) at 40× magnification. The cells were divided into four categories as follows: living (normal green nucleus), early apoptotic (bright green nucleus with condensed or fragmented chromatin), late apoptotic (orange-stained nuclei with chromatin condensation or fragmentation), and necrotic cells (uniformly orange-stained cell nuclei). In each experiment, >300 cells/samples were counted to calculate the percentage of apoptotic cells.

### Apoptosis assessment

Cells were irradiated with a 6 MV X-ray beam at a dose of 2 Gy and collected after 30 min of culture. Then, the samples were stained with 5 μl Annexin V PE and 5 μl 7-aminoactinomycin D (BD Pharmingen, U.S.A.), according to the manufacturer’s instructions. Analysis was carried out immediately on an FACScan flow cytometer (BD Biosciences). All samples were assessed in triplicate.

### Cell Counting Kit-8 assay

Cell viability was determined using Cell Counting Kit-8 (CCK-8) assay after exposure to different doses of X-ray IR. Briefly, cells were seeded in 96-well plates at a density of 3 × 10^3^ cells/well and allowed to attach overnight. Then, cells were exposed to IR with a 6 MV X-ray beam at 2 Gy and cultured for 6 days. After treatment, cells were incubated daily for 1 h with 10 μg/ml CCK-8 solution (Dojindo, Japan) in a humidified chamber containing 5% CO_2_ at 37°C. Absorbance was measured on a microplate reader (Bio-Rad) at 450 nm. Each group was assessed in five replicate wells and all experiments were conducted in triplicate. Cell survival was calculated using the following formula: survival rate (%) = OD/OD 0 h × 100%.

### Colony formation assay

Colony formation assays were performed to assess the radiosensitivity of cells after IR. Suspensions containing 200, 400, 800, 1600 and 3200 cells were seeded into five of the six-well plates and exposed to 0, 2, 4, 6 or 8 Gy (2 Gy per fraction), respectively, with a 6 MV X-ray beam from an Elekta linear accelerator (Precise 1120; Elekta Instrument AB, Stockholm, Sweden) at a dose rate of 220 cGy/min. The cells were incubated for 7 days until colony appearance. Colonies were fixed for 15 min with carbinol and stained for 30 min with 0.1% Giemsa (AppliChem, Germany). Colonies containing >50 cells were counted. All experiments were performed three-times.

### Comet assay

The OxiSelect™ Comet Assay Kit was used according to the manufacturer’s instructions. Briefly, cells were harvested by scraping and centrifugation (700 × ***g***, 2 min) and then washed with PBS. Cell suspensions were mixed with liquefied Comet Agarose at a 1:10 ratio (v/v) and pipetted onto an OxiSelect Comet Slide (75 μl/well). After a 15-min embedding step (4°C, dark, horizontal position), cells were lysed (25 ml lysis buffer/slide, 30-min incubation, 4°C, dark, horizontal position) and treated with an alkaline solution (25 ml/slide, 30 min, 4°C, dark) to relax and denature the DNA. Finally, the samples were electrophoresed in a horizontal chamber (300 mA for 30 min) to separate intact DNA from damaged fragments. Samples were then washed with sterile MilliQ water, treated with 70% cold ethanol for 5 min, air-dried, stained with the DNA dye DAPI (100 μl/well), and viewed under an epifluorescence microscope using a DAPI filter (Thornwood, NY, U.S.A.).

### Subcutaneous tumor model

5-8F cells (5 × 10^6^ cells in 100 μl PBS) were injected subcutaneously into the right flank of male nude mice. Tumor volume was monitored and calculated using the equation V (mm^3^) = a × b^2^/2, where a is the largest diameter and b is the perpendicular diameter. When palpable tumors reached a volume of 150–250 mm^3^, mice were subjected to radiation with an Elekta 6-MV photon linear accelerator. Before IR, each mouse was anesthetized with 0.6% pentobarbital (40 mg/kg) and shielded by a lead box with only the xenograft tumor exposed. Five fractions of 2 Gy were delivered every 2 days for a total dose of 10 Gy with a dose rate of 1 Gy/min. After the final IR treatment, mice were observed for 14 consecutive days. When the 14-day protocol was completed, tumor weight was measured and the tumor growth inhibitory rate calculated.

### Histological examination

Tissue samples were fixed in 4% paraformaldehyde, dehydrated and embedded in paraffin for staining with Hematoxylin–Eosin. Tissue blocks were sectioned, examined under an Olympus BX51 microscope, and photographed using an Olympus DP71 digital camera.

### Western blot analysis

Proteins were extracted after the cells were irradiated. Quantified cell lysates were separated on 8–12% SDS polyacrylamide gels and electroblotted onto polyvinylidene membranes. After blocking for 0.5 h, the membranes were incubated sequentially at 4°C overnight with primary antibodies against γ-H2AX (1:1000; #L7543, Proteintech), ATM (pSer1981) (1:500; #AF4120, Affinity), ATR (pSer428) (1:1000; #DF7512, Affinity), CHK1 (pSer345) (1:1000; #2348, CST), CHK2 (Thr68) (1:1000; #2197, CST), CCNB1 (1:1000; 220491, zenbio), CDC2 (1:1000; #9111, CST) and β-tubulin (1:20,000; #FD064, FD technology). The membranes were washed and incubated for 1 h at room temperature with a secondary antibody (1:15,000; #A0208, Beyotime Biotechnology, Shanghai, People’s Republic of China). Western blotting bands were visualized with the eECL Western Blot Kit (CWBIO Technology) and images were captured with the ChemiDoc™ CRS þ Molecular Imager (Bio-Rad).

### Statistical analysis

Data are presented as the mean ± standard deviation from ≥3 independent experiments. Differences were considered statistically significant at *P*<0.05 (Student’s *t*-test for two groups, one-way analysis of variance for multiple groups, and a parametric generalized linear model with random effects for tumor growth). Calculations were performed using SPSS 19 software (SPSS, Inc., Chicago, IL, U.S.A.).

## Results

### EBV-miR-BART8-3p promotes proliferation and inhibits apoptosis independently of IR

**Figure 1:**
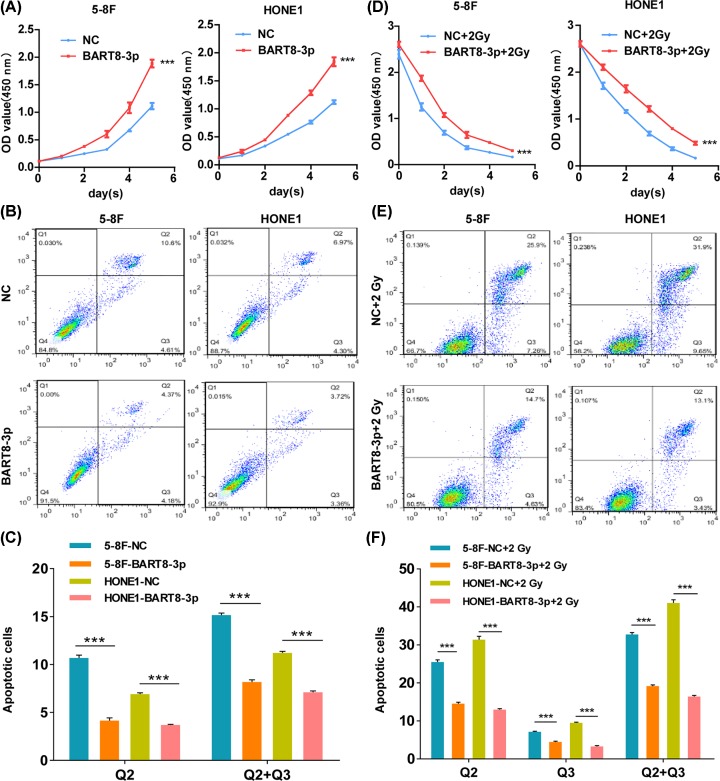
EBV-miR-BART8-3p promotes proliferation and inhibits apoptosis (**A,D**) The proliferative ability of NPC cells was determined using the CCK-8 assay in the presence (**D**) or absence (**A**) of IR at a single dose of 2 Gy. (**B,E**) The rate of apoptosis was determined by flow cytometry in the presence (**E**) or absence (**B**) of IR at a single dose of 2 Gy. (**C,F**) Bar graphs show total apoptosis under all conditions: Q2 indicates early apoptosis and Q3 indicates late apoptosis (^***^P<0.001).

EBV-miR-BART8-3p-transfected cell lines were generated as shown in Supplementary Figure S1A. The results of the CCK-8 assay indicated that EBV-miR-BART8-3p increased proliferation of NPC cells compared with the negative control (NC) cells (Figure 1A). Flow cytometry analysis showed that the rate of apoptosis in cells overexpressing EBV-miR-BART8-3p was lower than that in NC cells (Figure 1B); this was confirmed by quantitative analysis (Figure 1C). EBV-miR-BART8-3p overexpression increased NPC cell proliferation under IR conditions (Figure 1D). Cell apoptosis was decreased 11.2% (5-8F) and 18.8% (HONE1) (Figure 1E,F), and this was confirmed by AO and EB double staining (Supplementary Figure S1B).

### EBV-miR-BART8-3p promotes radioresistance of NPC *in vitro*

**Figure 2:**
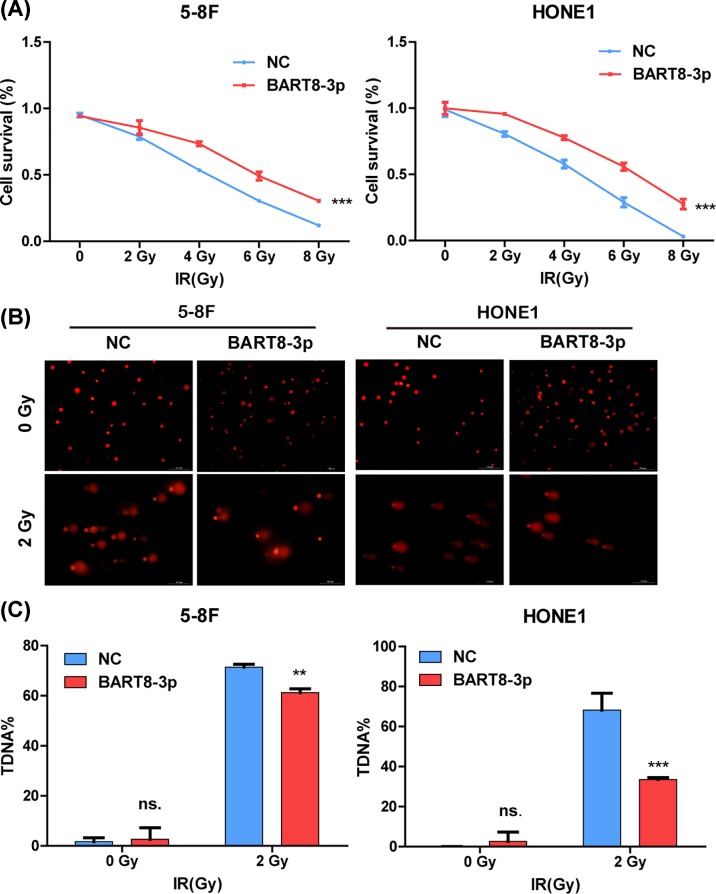
EBV-miR-BART8-3p promotes NPC radioresistance *in vitro* (**A**) Inhibition of cell proliferation by IR at 0, 2, 4, 6, or 8 Gy was determined in a colony formation assay. (**B**) Percentage of DSBs in response to 0 or 2 Gy of IR, as determined in a comet assay. (**C**) Bar graphs represent the percentage of DSBs of NPC cells. (^**^P<0.01, ^***^P<0.001).

Stably transfected cell lines were constructed (Supplementary Figure S2A,B) and colony formation and comet assays were performed to confirm the relationship between radioresistance and EBV-miR-BART8-3p (Figure 2). The colony formation assay showed that survival of stably transfected NPC cells decreased in response to IR in a dose-dependent manner (Supplementary Figure S2C), and the rate of decrease was lower in EBV-miR-BART8-3p stably transfected cells than in the NC group. Cell survival in the NC group decreased to almost zero, whereas it was higher in the experimental group in response to doses up to 8 Gy (Figure 2A). The results of the comet assay showed that the percentage of DNA DSBs was higher in the NC group than in the experimental group after exposure to 2 Gy of IR (Figure 2B), suggesting that overexpression of EBV-miR-BART8-3p attenuated IR-induced DSBs. Taken together, these results indicate that EBV-miR-BART8-3p promotes radioresistance *in vitro*.

### EBV-miR-BART8-3p promotes radioresistance of NPC *in vivo*

**Figure 3:**
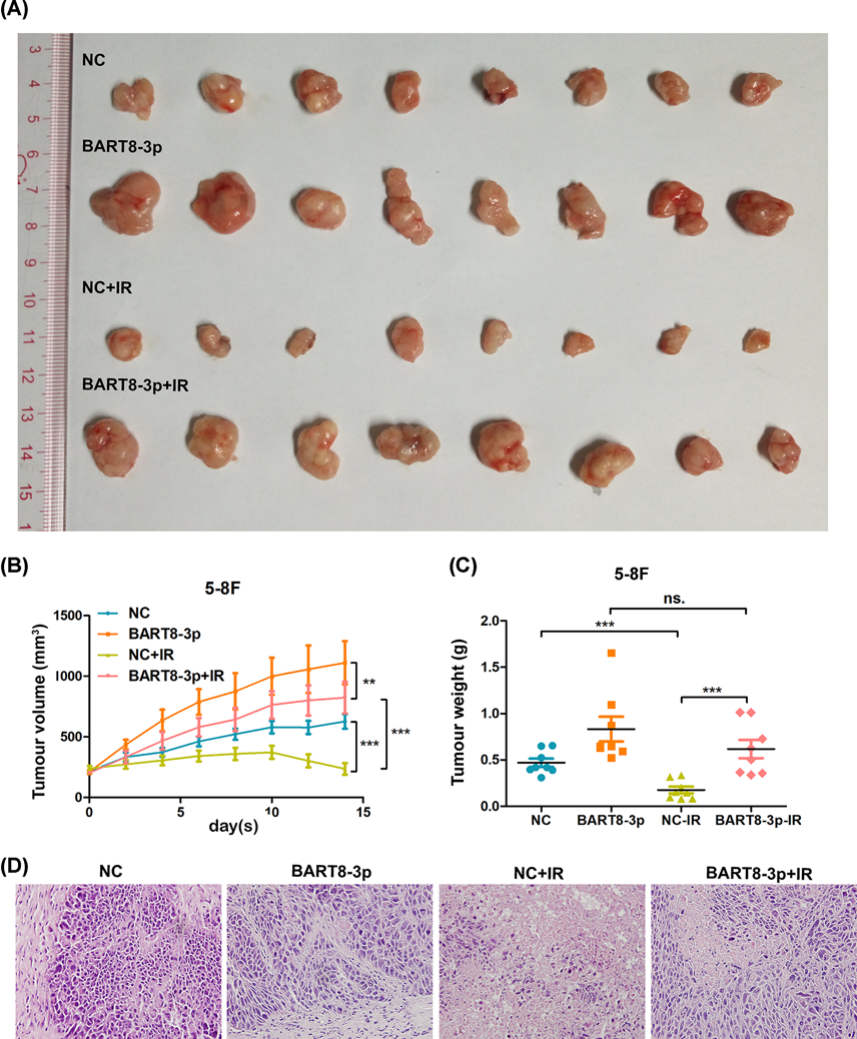
EBV-miR-BART8-3p promotes NPC radioresistance *in vivo* (**A**) Xenograft tumors generated by subcutaneous implantation of 5-8F cells were excised on Day 14 after reaching a volume of 200 mm^3^. The NC-IR and BART8-3p-IR groups were exposed to 2 Gy IR every 2 days and the volume (**B**) and weight (**C**) of tumors are shown. Histologic evaluation of tumor structure (**D**). (^**^P<0.01, ^***^P<0.001; *n*=8).

A tumor xenograft model in nude mice was generated by subcutaneous implantation of 5-8F cells. On Day 14, tumor volume in the experimental group was larger than that in the NC group after exposure to 0 and 2 Gy IR (Figure 3A). Quantification of tumor volume and weight showed that IR decreased the rate of tumor growth in the NC and experimental groups (Figure 3B). However, the rate of tumor growth was higher in the experimental group than in the NC group (Figure 3C). The results of histologic evaluation of tumor tissues from the different groups are shown in Figure 3D. These results suggest that EBV-miR-BART8-3p promotes radioresistance of NPC *in vivo*.

### EBV-miR-BART8-3p inhibits IR-induced DSBs

**Figure 4:**
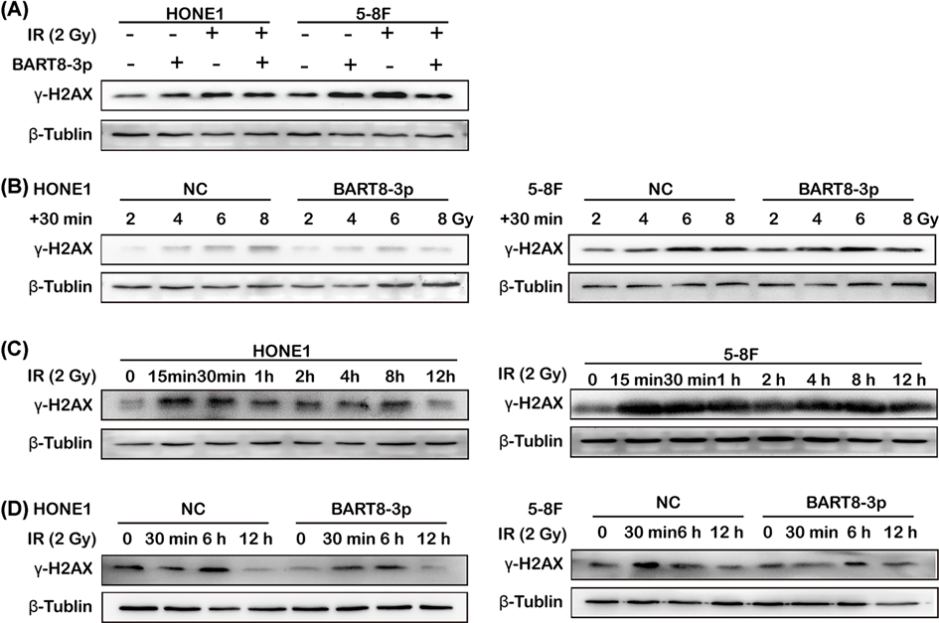
EBV-miR-BART8-3p inhibits IR-induced DSBs Western blot analysis of γ-H2AX expression in response to 0 or 2 Gy IR (**A**) or 0, 2, 4, 6 and 8 Gy IR (**B**). Western blot analysis of γ-H2AX expression in response to 0–12 h of IR at 2 Gy in the 5-8F cells and HONE1 cells (**C**). Western blot analysis of γ-H2AX expression in response to 0 min, 30min, 6 h, 12 h of IR at 2 Gy in the two groups (**D**).

Detection of γ-H2AX was used to investigate formation of DNA DSBs. The results of Western blot analysis showed that IR up-regulated γ-H2AX expression in the NC and experimental groups, but that the degree of up-regulation was lower in the experimental group than in the NC group (Figure 4A). Overexpression of EBV-miR-BART8-3p suppressed IR-induced DSBs, as shown by the lower degree of γ-H2AX up-regulation in response to increasing doses of IR in the experimental group than in the NC group (Figure 4B). In cells exposed to a constant dose of 2 Gy, γ-H2AX in both cell lines increased in the first 15 min, followed by a decrease at 12 h (Figure 4C), which is consistent with the pattern of γ-H2AX expression associated with DNA damage and DSB repair. γ-H2AX expression followed the same pattern in both groups, although it was lower in the experimental group than in the NC group at each time point (Figure 4D). These results indicate that EBV-miR-BART8-3p inhibits formation of DSBs induced by IR.

### EBV-miR-BART8-3p activates ATM/ATR signaling pathway for DNA repair

**Figure 5:**
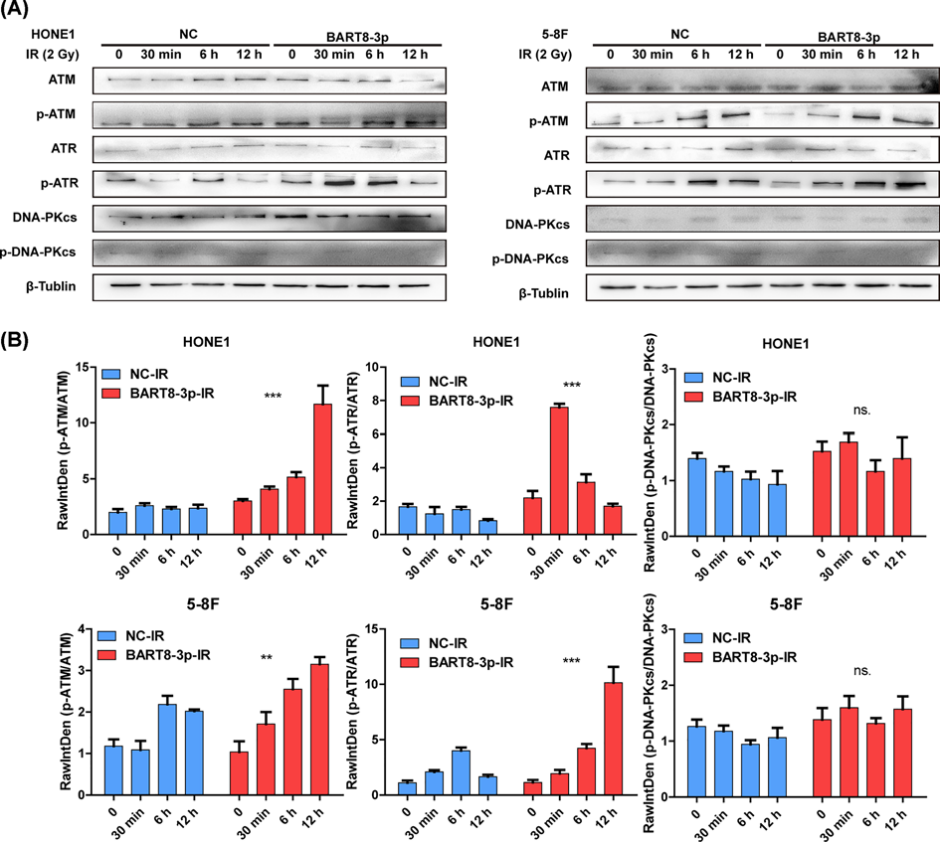
EBV-miR-BART8-3p activates ATM/ATR (**A**) Western blot analysis of p-ATM/ATM, p-ATR/ATR and p-DNA-PKcs/DNA-PKcs in response to 0–12 h of IR at 2 Gy in the two groups. (**B**) Bar graphs represent the gray scale of p-ATM/ATM, p-ATR/ATR and p-DNA-PKcs/DNA-PKcs. (^**^P<0.01, ^***^P<0.001).

ATM, ATR are important factors for DSB repair. Assessment of p-ATM and p-ATR expression showed that IR increased ATM and ATR activity in both groups in a time-dependent manner, and that the activity was higher in the experimental group than in the NC group. By the way, DNA damage repair protein and cell cycle regulatory protein mediated by ATM/ATR were changed. The expression of p-CHK2/p-CHK1 and CCNB1-CDK1 are up-regulated with the activation of ATM/ATR. (Figure 5A and Supplementary Figure S5). Western blot analysis confirmed that the increase in ATM and ATR activity was higher in the experimental group than in the NC group (Figure 5B). Taken together, these results indicate that EBV-miR-BART8-3p activates ATM/ATR signaling pathway during the DSBs repair process.

### KU60019/AZD6738 inhibits ATM/ATR activity

**Figure 6:**
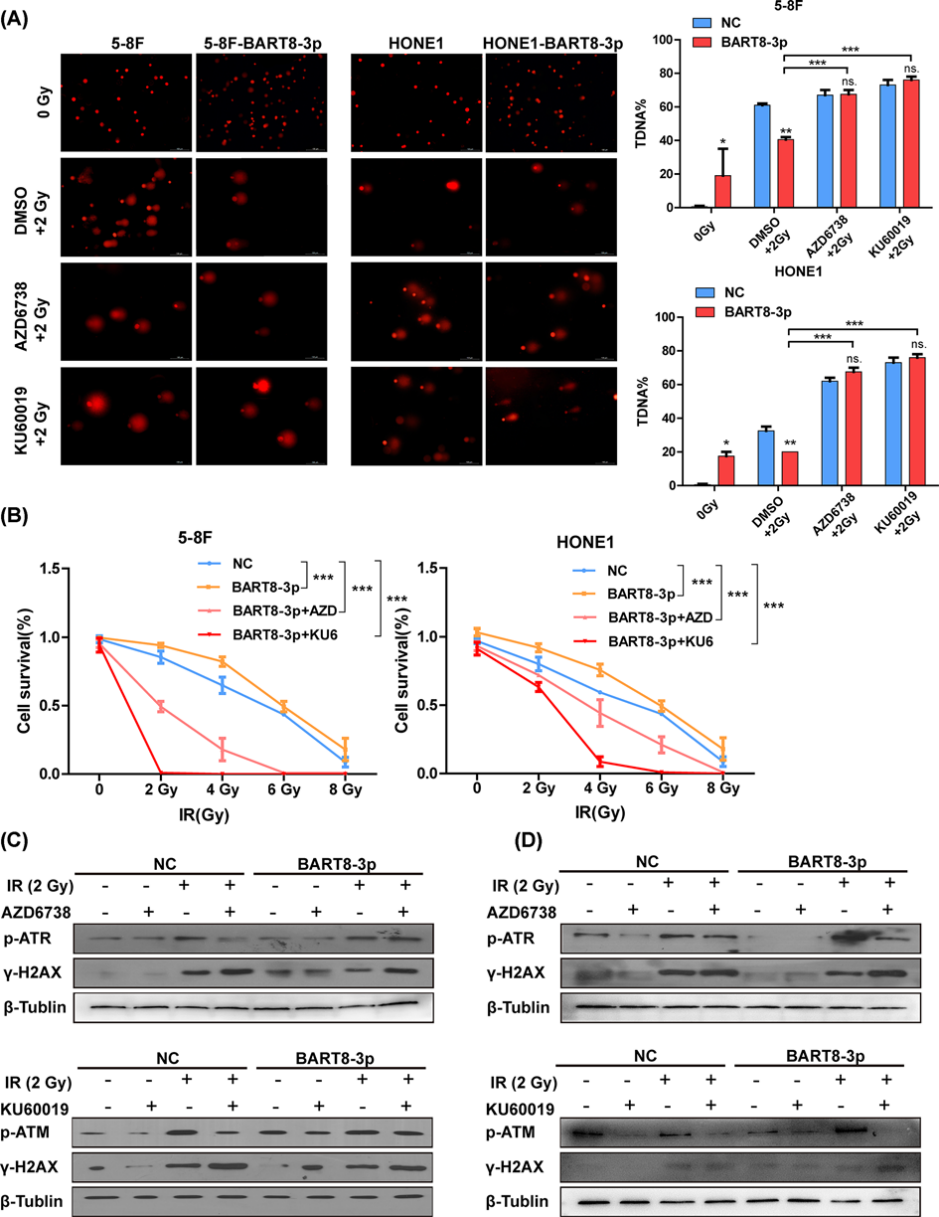
KU60019/AZD6738 inhibit ATM/ATR activation (**A**) Formation of DSBs in response to treatment with KU60019 or AZD6738 in the presence of 2 Gy IR, as determined in a comet assay. (**B**) Cell survival in response to treatment with KU60019 or AZD6738 in the presence of 2 Gy IR, as determined in a colony formation assay. (**C**) Western blot analysis of p-ATR/γ-H2AX and p-ATM/γ-H2AX responses to treatment with KU60019 or AZD6738 under IR conditions. (^*^P<0.05,^ **^P<0.01, ^ ***^P<0.001).

The results of the comet assay showed that treatment with KU60019 or AZD6738 promoted formation of DSBs in both groups under IR conditions (Figure 6A). The percentage of DSBs in the 5-8F cell line was comparable with that in the NC group, whereas the percentage of DSBs in the HONE1 cell line was higher than that in the NC group (Figure 6A). Colony formation assays showed that survival of cells treated with KU60019 or AZD6738 was lower in the experimental group than in the NC group (Figure 6B). Detailed data from the colony formation assays are shown in Supplementary Figures S3 and S4. Expression of p-ATR, p-ATM, decreased and γ-H2AX increased in response to treatment with AZD6738/KU60019 under 2 Gy IR. (Figure 6C,D).

## Discussion

The results of the present study show that EBV-miR-BART8-3p promotes radioresistance of NPC *in vivo* and *in vitro*. Irradiation up-regulated γ-H2AX, indicating an increase in DSB, whereas ATM/ATR activation induced by EBV-miR-BART8-3p inhibited this process.

Lots of miRNAs contribute to the radioresistant of different carcinoma. In the context of esophageal cancer, recent studies have demonstrated clinical correlations of sets of miRNAs with the outcome of radiotherapy, where expression of one set of miRNAs promotes the development of radioresistance, while another set sensitizes esophageal cancer cells to radiation therapy [15]. Preoperative radiotherapy has become a standard method for the treatment of patients with locally advanced CRC. A recent study demonstrated that *miR-198, miR-765, miR-630, miR-371-5p, miR-575, miR-202* and *miR-513a-5p* may be used for predicting the response of CRC to preoperative radiotherapy [16]. Following IR, several miRNAs were indicated changed. Radical radiotherapy is the first choice of primary treatment, while radiotherapy still has many obstacles to overcome, like radioresistance[17]. Few miRNAs related to radioresistant of NPC were reported. *miR-483-5p* decreases the radiosensitivity of nasopharyngeal carcinoma cells by targeting DAPK1 [18]. *miR–495* enhances the efficacy of radiotherapy by targeting GRP78 to regulate EMT in nasopharyngeal carcinoma cells [19]. Besides, EBV-associated miRNAs are known to modulate multiple viral and human mRNAs in NPC. EBV-miR-BART4 affects growth and apoptosis in NPC cells exposed to IR, implying a possible role for EBV-miR-BART4 in the radioresistance of NPC [13]. This is consistent with the present results. Overexpression of EBV-miR-BART8-3p resulted in the decreased apoptosis and increased proliferation of NPC cell exposed to IR *in vitro*. Besides, overexpression of EBV-miR-BART8-3p was not as successful as the NC group in reducing tumor volume and weight with radiotherapy *in vivo*. What’s confusing to us is that there is no difference in tumor weight between EBV-miR-BART8-3p and EBV-miR-BART8-3p-IR. We suspect that radiation treatment increases tissue necrosis, fibrosis and density [20]. May be this is the most important reason why the weight and volume results are inconsistent. While there is no difference in tumor weight between EBV-miR-BART8-3p and EBV-miR-BART8-3p-IR, volume reduction and well-defined boundaries mean that radiotherapy is effective.

γ-H2AX is a marker of DSBs that is used to monitor DNA damage and repair. Changed γ-H2AX expression in cells suggested relationship between EBV-miR-BART8-3p and DSBs (the most common way of DNA damage caused by IR)/DSBs repair in NPC under IR conditions. Early in the DNA damage response, ATM phosphorylates histone H2AX at serine 139 on the C-terminus in multiple chromatin sites flanking DNA DSBs, thereby generating γ-H2AX [21]. ATM is an essential molecule in the homologous recombination pathway, as it responds immediately to DNA damage and activates several downstream effectors to interrupt the cell cycle and stop DNA replication [22]. ATR is a member of the phosphatidylinositol 3-kinase-like kinase family, which functions together with ATM as a central regulator of cellular responses to DNA damage [23]. In addition, ATM/ATR activates downstream CHK2/CHK1, further regulating the DNA repair process[24]. In the present study, EBV-miR-BART8-3p and EBV-miR-BART4 had similar effects on radioresistance of NPC, whereas they played different roles in regulation of ATM/ATR during this process. EBV-miR-BART8-3p activated ATM/ATR signaling pathway, thereby inducing NPC radioresistance by DSBs repair under IR conditions. The regulatory ability of EBV-miR-BART8-3p is affected by IR or possibly by the synergism of EBV-miR-BART8-3p and IR. This latter phenomenon could not be confirmed, and additional studies are necessary to clarify this mechanism. Several signaling molecules were regulated by ATM/ATR, while the most important set of molecules were cell cycle-related Cyclin/CDK compounds including CycB/CDK1, CycA/CDK1, CycH/CDK7, CycA/CDK2, CycE/CDK2, CycD/CDK4, 6. Radiosensitivity was enhanced specifically through inhibition of CDK1, which prolonged G2/M arrest, delayed DSBs repair and increased apoptosis [25,26]. In our research, up-regulation of p-ATM/p-CHK2, p-ATR/p-CHK1 and CycB/CDK1 by EBV-miR-BART8-3p in NPC may, at least partly, explain the high radioresistance of this deadly cancer.

KU-60019 is a specific ATM kinase inhibitor that sensitizes tumor cells to radiation in the low micromolar range. Radiosensitization is related to the ability of KU-60019 to inhibit ATM phosphorylation targets and disrupt cell cycle checkpoints, inhibit DNA repair and promote cell death. Inhibition of basal AKT phosphorylation by KU-60019 affects cell growth independently of IR [27]. The relationship between KU60019 and AKT will be explored in our follow-up study. AZD6738, a highly selective and potent inhibitor of ATR kinase activity that is both orally active and bioavailable has the same effect as KU-60019. AZD6738 induces ATM kinase-dependent DNA damage signaling and potentiates cell killing by cisplatin [28]. The present results suggest a potential effect of KU-60019/AZD6738 on the response of NPC to IR, thereby providing new ideas for clinical treatment of NPC. ATM/ATR inhibitors could be developed to improve the response of NPC to radiotherapy in the future.

## Availability of data and materials

The datasets used and/or analyzed during the current study are available from the corresponding author on reasonable request.

## Supporting information

**Supplementary Figure S1:** 

**Supplementary Figure S2:** 

**Supplementary Figure S3:** 

**Supplementary Figure S4:** 

**Supplementary Figure S5:** 

## Abbreviations

AO: acridine orange
ATM: ataxia telangiectasia mutated
ATR: ataxia telangiectasia mutated and Rad3-related
BART: BamHI fragment A rightward transcript
BHRF1: BamHI fragment H rightward reading frame 1
CCK-8: Cell Counting Kit-8
cDNA: complementary DNA
CRC: colorectalcancer
DAPI: Dihydrochloride
DAPK1: Death assosiated protein kinase 1
DSB: DNA double-strand break
EB: ethidium bromide
EBV: Epstein-Barr virus
EMT: Epithelial-Mesenchymal transition
GFP: Green fluorescent protein
IR: irradiation
miRNA: microRNA
NC: negative control
NPC: nasopharyngeal carcinoma
OD: optical density
p-ATM: phosphor-ATM
p-ATR: phosphor-ATR
qRT-PCR: Real-time Polymerase Chain Reaction
RPMI: Roswell Park Memorial Institute
