# Expression of Concern: EBV encoded miRNA BART8-3p promotes radioresistance in nasopharyngeal carcinoma by regulating ATM/ATR signaling pathway

**DOI:** 10.1042/BSR20190415_EOC

**Published:** 2026-05-06

**Authors:** Longmei Cai, Xiaohan Zhou, Jialing Zheng, Ying Tang, Yanling Lin, Lingzhi Wang, Ye Li, Chengdong Liu, Dehua Wu

**Affiliations:** Department of Radiation Oncology, Nanfang Hospital, Southern Medical University, Guangzhou, China

**Keywords:** ATM/ATR, DSBs, EBV-miR-BART8-3p, NPC, radioresistance

*Bioscience Reports* has been made aware of potential issues surrounding the scientific validity of this paper and is hence issuing a permanent Expression of Concern to notify readers. In Figure 5A, the HONE1 p-DNA-PKcs bands appear to be the same as the 5-8F p-DNA-PKcs bands.

The authors have cooperated with the journal's investigation and provided raw data; however, some uncertainty remains regarding the data provided. As such, the article will remain published but with an associated Expression of Concern.

