# Supplementary figures and images for: EBV encoded miRNA BART8-3p promotes radioresistance in nasopharyngeal carcinoma by regulating ATM/ATR signaling pathway
Source: Biosci Rep. 2019 Sep 13;39(9):BSR20190415. doi: 10.1042/BSR20190415 (PMC6744588; doi:10.1042/BSR20190415)

Supplementary Fig. 1

A

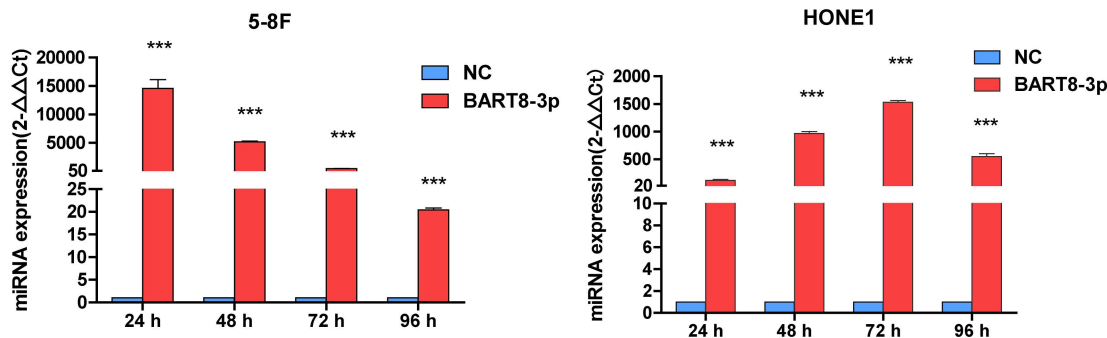

B

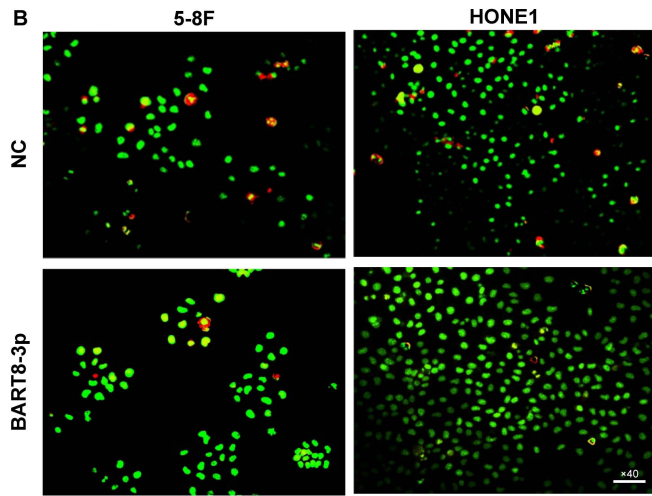

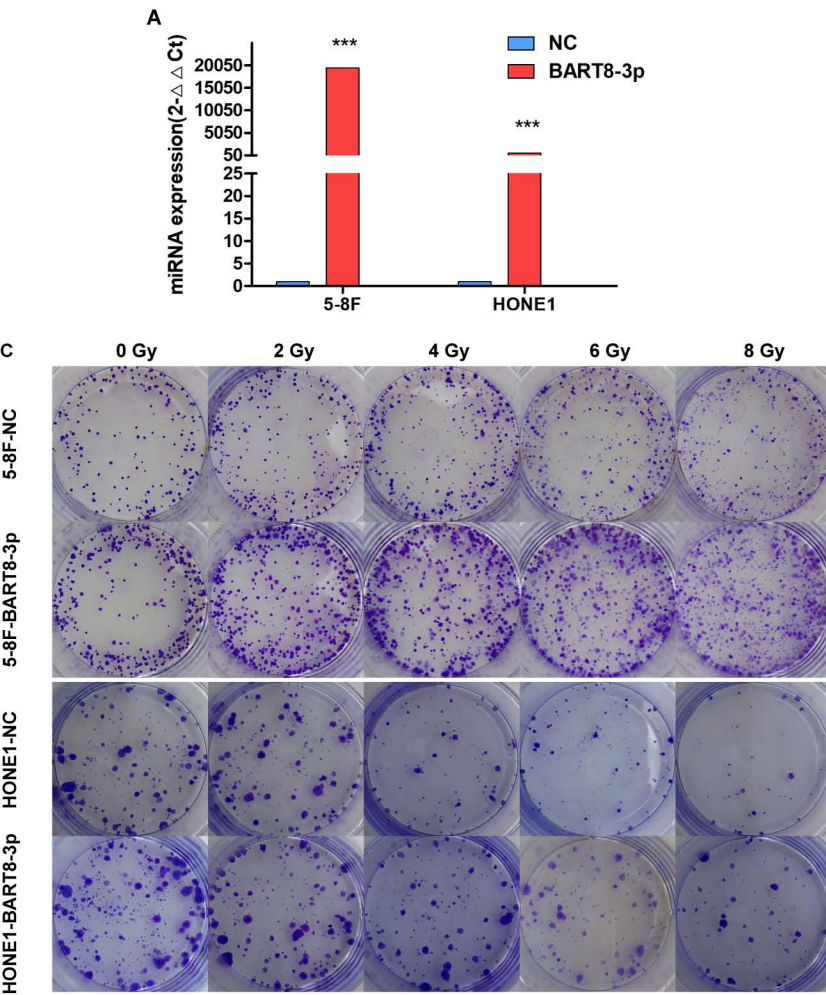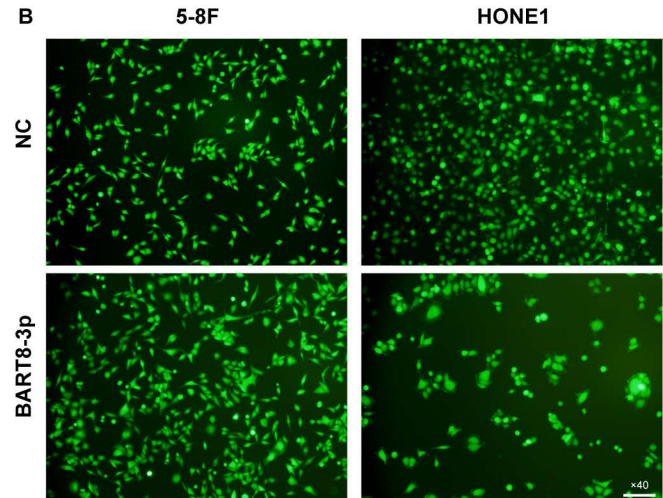

Supplementary Fig. 3

HONE1

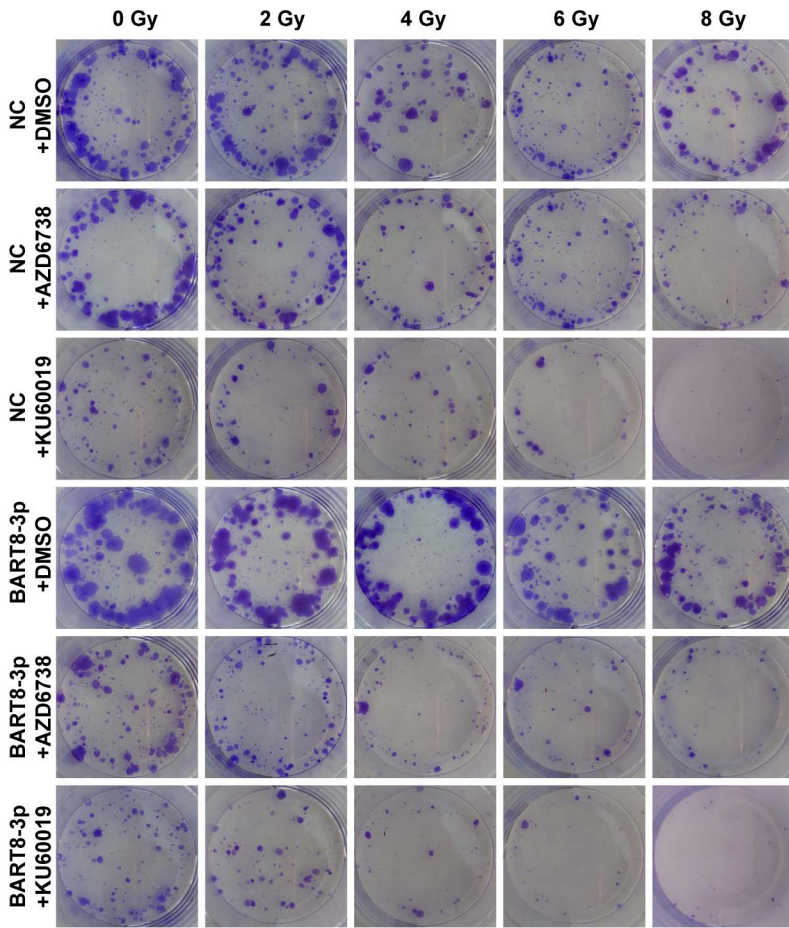

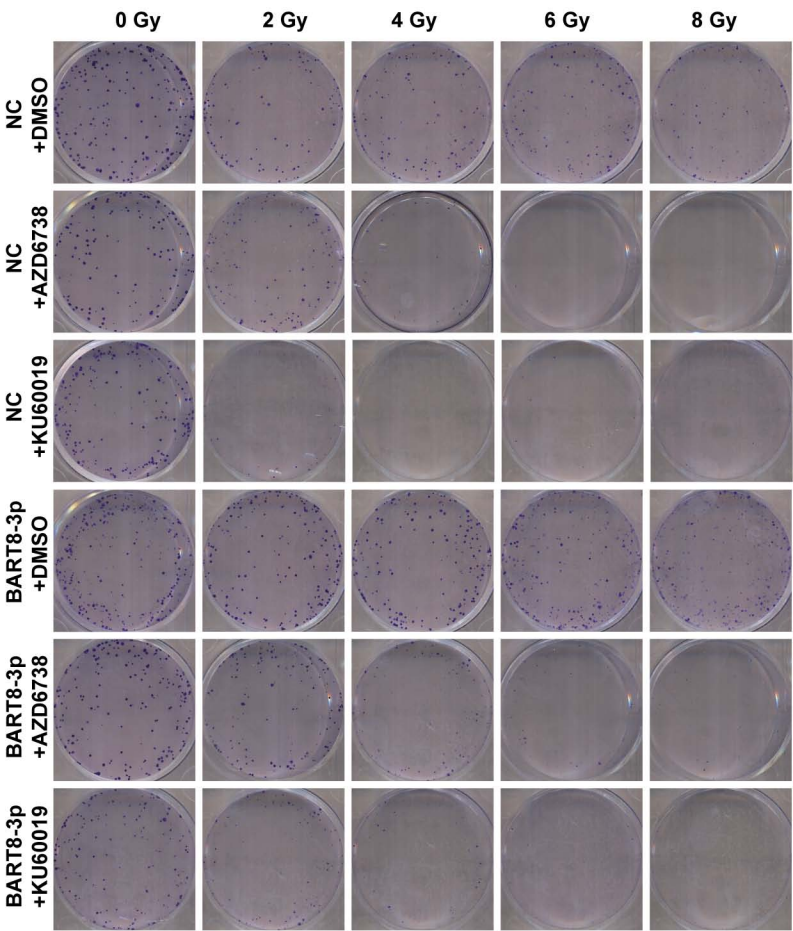

**Supplementary Fig.5**

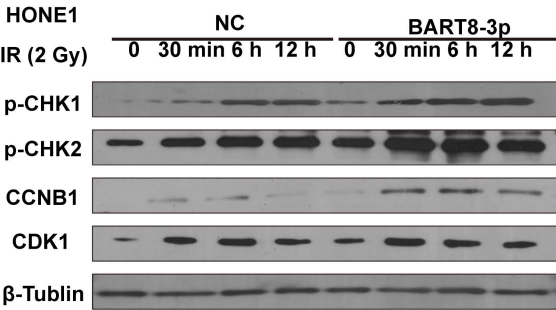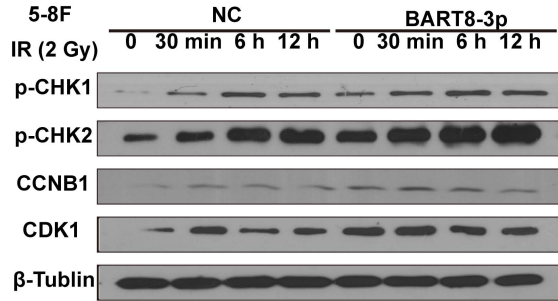

Supplement: Supplementary file 1 [file bsr20190415_Supp1.pdf]
